# Potentially Avoidable Spine Transfers to a Level I Trauma Center: A Five-Year Retrospective Analysis

**DOI:** 10.5811/westjem.47485

**Published:** 2026-06-28

**Authors:** Clifford M. Marks, Ryan C. Burke, Cody Rasner, Martina Stippler, Carlo L. Rosen

**Affiliations:** * Harvard Medical School, Beth Israel Deaconess Medical Center, Department of Emergency Medicine, Boston, Massachusetts; † Harvard Medical School, Beth Israel Deaconess Medical Center, Department of Neurosurgery, Boston, Massachusetts

## Abstract

**Background:**

Emergency physicians frequently transfer patients with spine pathology to tertiary-care centers, although research suggests many are discharged without requiring a procedure or specialized imaging. Such transfers consume resources and remove patients from their families and communities. Better understanding these transfers might help identify which patients can receive care without transfer.

**Methods:**

This was a retrospective cohort study of transfers for spine evaluation to an urban, tertiary-care, Level I trauma center from October 1, 2017–October 1, 2022. Transfers were defined as necessary if the patient a) went to the operating room (OR) within 12 hours of arrival, b) had a neurologic magnetic resonance image (MRI), c) was admitted to an intensive care unit from the emergency department (ED), or d) was admitted to either neurology or a surgical service (including neurosurgery and orthopedics, which share spine coverage in the study hospital).

**Results:**

The study included 1,918 transfers with a spine evaluation, of which 617 (32.2%) were deemed potentially avoidable. Just 3.3% of all transfers went to the OR within 12 hours of receiving ED arrival, 45.7% had a neurologic MRI at the receiving hospital, and 36.6% were admitted to a neurology or a surgical service. For all transfers, 17.7% were discharged directly from the ED, 16.8% were placed in observation and then discharged, 6.8% were placed in observation and then admitted, and 58.7% were admitted to inpatient from the ED. The average ED length of stay for necessary spine transfers was 15.4 hours vs 16.6 hours for potentially avoidable transfers (P = .07). Compared to the overall rate of necessary transfers (67.8%), patients with the following conditions had a decreased incidence of necessary transfers: cervical spine fractures (62% necessary, P = .008), lumbar spine/sacrum/coccyx fractures (53.5% necessary, P < .001), and disc disorders (51.4% necessary, P = .003). Patients diagnosed with cervical spinal cord lesions (91.7% necessary, P = .01) had an increased incidence of necessary transfers.

**Conclusion:**

Nearly one-third of spine evaluation transfers were potentially avoidable. Patients transferred for spine evaluation experienced prolonged ED stays, creating challenges for both receiving hospitals and patients. Further research is needed to prospectively identify patients who do not require tertiary care and to study methods for managing them in community settings.

## INTRODUCTION

Tens of thousands of patients in the United States are transferred to tertiary-care hospitals annually for spine evaluation.^1^ While a subset do require surgical care available only at tertiary sites, earlier research suggests that as many as 79% of patients transferred for isolated spine pathology were discharged directly from the emergency department (ED), some with a brace and others with no treatment at all.^2^ These potentially avoidable transfers can involve real harm from a social and patient perspective. Both patients and the healthcare system as a whole pay thousands and sometimes tens of thousands of dollars for each transfer, particularly when air medical transport is involved.^2^,^3^ In many cases, patients are sent far from their communities, making it difficult for family members to provide needed support.^4^ Moreover, the long lengths of stay associated with such cases can further burden tertiary EDs already plagued by a boarding crisis.

While reducing unnecessary transfers is a laudable goal, determining prospectively which patients are unlikely to benefit from transfer remains a central challenge. Pilot programs for spine telehealth evaluation suggest remote assessments can be quite accurate, but more research is needed to safely develop and study such interventions. To that end, we performed a five-year retrospective analysis of spine transfers to a U.S. Level I trauma center—the largest such study to date. Our objectives were to 1) characterize the magnitude of potentially avoidable spine transfers to a Level I trauma center over a five-year period; 2) identify patient and clinical characteristics associated with potentially avoidable transfers; and 3) determine which diagnostic categories are most strongly associated with unnecessary transfers to inform future intervention strategies.

## METHODS

### Study Design

We conducted a retrospective cohort study of patients transferred for spine service evaluation to an urban Level 1 trauma center, where the ED sees 55,000 patients annually. The cohort included all visits from patients ≥ 18 years of age who were transferred between October 1, 2017–October 1, 2022 and evaluated by the spine consult service in the ED. The five-year study period was chosen to capture sufficient cases for meaningful analysis. No formal sample-size calculation was performed as this was a descriptive study aimed at characterizing all spine transfers in the study period, rather than one intended to test specific hypotheses with a predetermined effect size. Transferred patients with a spine consult in the ED were defined as transferred patients for whom the emergency physician in the receiving ED ordered a spine consult. This cohort, thus, included both transfers solely for spine evaluation and polytrauma transfers for which the spine service was consulted.

We did not collect data about the transferring clinicians or their working diagnosis at the time of transfer. A visit was excluded from this study if the patient died in the ED or if the ED disposition was eloped or left against medical advice, as these criteria rely on physician decisions to operate, admit, or order imaging and may not be reflected for patients who did not complete their ED workup. As this was an analysis of a database of patients, we did not perform manual chart review and, thus, did not report on abstractor training, use of abstractor forms, or inter-rater reliability.^5^ This study is reported in accordance with the Strengthening the Reporting of Observational Studies in Epidemiology Statement^6^ (Supplemental Table 1).

**Population Health Research Capsule**

What do we already know about this issue?

*Many patients transferred to tertiary centers for spine evaluation are discharged without surgery or specialized imaging, suggesting some transfers may be unnecessary*.

What was the research question?

*What proportion of spine transfers to a Level I trauma center were potentially avoidable over five years?*

What was the major finding of the study?

*Of 1,918 spine transfers, 617 (32.2%) were potentially avoidable*.

How does this improve population health?

*The significant proportion of potentially avoidable transfers for spine evaluation represents a key opportunity to reduce costs, ED crowding, and harm to patients*.

### Variables

We employed criteria previously used in a study of neurosurgical transfers to differentiate necessary spine transfers from potentially avoidable transfers.^7^ A transfer was deemed necessary if the patient a) went to the operating room (OR) within 12 hours of arrival to the ED; b) a neurologic magnetic resonance image (MRI), defined as an MRI of the brain, neck or spinal cord, was conducted in the ED; c) the patient was admitted to intensive care unit (ICU)-level care from the ED; or d) the patient was admitted to either neurology or a surgical service (including neurosurgery or orthopedics, which share spine coverage in the study hospital). A transfer that did not meet any of the above criteria was categorized as potentially avoidable.

We retrospectively collected demographic data, Emergency Severity Index (ESI) level, disposition, *International Classification of Diseases, 10^th^ Rev*, (*ICD-10*) codes, and neurologic MRI ordering information in the ED via electronic health record data abstraction. Missing data patterns were assessed but not imputed. We evaluated data completeness for key variables, with only records containing complete data for the primary outcome classification (necessary vs potentially avoidable transfer) included in the analysis. Drawing on a previously published categorization of spine injuries, we devised diagnostic categories (eg, “Cervical spine fracture” or “Spinal infection”) with relevant *ICD-10* codes assigned to each category^8^ (see Table 1). We then categorized each visit according to its *ICD-10* codes. Because some visits had multiple *ICD-10* codes, it was possible for one visit to be counted in more than one category. To ensure uniform coding practices regardless of patient disposition, analysis was restricted to *ICD-10* codes assigned by the emergency physician at the time of disposition decision.

### Statistical Tests

We summarized patient and clinical characteristics with frequencies and percentages, both overall and stratified by whether the transfer was classified as necessary or potentially avoidable. We assessed statistical differences between the two groups with a chi-square test for categorical variables and a *t*-test for continuous variables. A *P* value of < .05 was considered significant. A Benjamini-Hochberg adjustment for multiple comparisons was performed for the significance testing of diagnostic groupings. We performed all analyses in statistical analysis system SAS v9.4 (SAS Institute Inc, Cary, NC).

## RESULTS

There were 1,949 visits by adult patients transferred for spine service evaluation during the study period. Of those, 31 were excluded based on ED disposition, leaving 1,918 visits in our study. Data required for primary outcome classification were complete for all 1,918 included patients. The mean age for this cohort was 65 years (standard deviation 19 years) and 943 were women (49.2%). The cohort contained 1,556 White patients (81.1%), 112 Black patients (5.8%), 54 Hispanic/Latino patients (2.8%), 38 Asian patients (2.0%), 90 patients listed as “other” (4.7%), and 68 whose ethnicity was unknown (3.5%).

**Figure 1:**
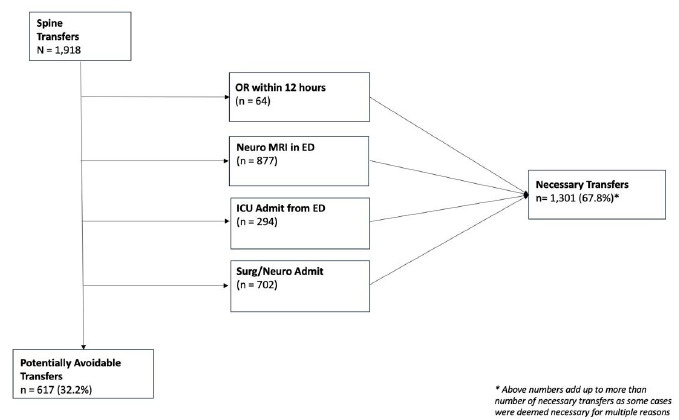
Categorization of needed vs. potentially avoidable transfers in a study of interhospital transfers for neurosurgical evaluation. *ED*, emergency department; *ICU*, intensive care unit; *MRI*, magnetic resonance image; *OR*, operating room.

Nearly one-third of transfers (617, 32.2%) were deemed potentially avoidable by our criteria. Sixty-four (3.3%) of all spine transfers went to the OR within 12 hours of arriving at the receiving hospital, 15.3% were admitted to the ICU from the ED, 45.7% had a neurologic MRI performed while in the ED, and 36.6% were admitted to neurology or to a surgical service (see Figure 1).

We present demographic and clinical characteristics for the study population overall and stratified by need for transfer in Table 2. Potentially avoidable transfers were significantly more likely to be female patients compared to needed transfers (57.7% vs 45.1%, *P* < .001). Potentially avoidable transfers were patients significantly older than those needing transfer (67 vs 65 years of age, *P* = .01). Race and ethnicity were statistically associated with transfer necessity (*P* = .009) but this significance disappeared when patients with unknown race/ethnicity were removed from the analysis (*P* = .77). Whether the patient was English speaking was not associated with transfer necessity (*P=*0.72). The ESI was significantly associated with needing to be transferred (*P* < .0001), with a higher proportion of ESI 1 in the needing transfer group (17.5% vs 5.2%) and a lower proportion of ESI levels 3–5 (18.2% vs 32.3%). A statistically higher proportion of visits needing transfer were self-pay (7.1% vs 2.8%, *P* < .001).

The overall cohort had a mean ED length of stay of 15.8 hours (SD 12.7 hours). Potentially avoidable transfers tended to spend longer in the ED than necessary transfers (mean 16.6 vs 15.4 hours), although this difference did not reach statistical significance **(***P* **=** .07).

Table 3 shows diagnostic categories for the transferred patients overall and stratified by need for transfer, as well as the probability a patient in each diagnostic grouping needed transfer. Patients with diagnoses in the following categories were significantly more likely to have been potentially avoidable transfers: Cervical spine fractures (22.4% of potentially avoidable transfers vs 17.3%, *P* **=** .008), lumbar spine/sacrum/coccyx fractures (15.1% vs 8.2%, *P* < .001), back pain (23.3% vs 17.4%, *P* = .002), and disc disorders (5.5% vs 2.8%, *P* = .003). Patients with diagnoses designated as cervical spinal cord lesions (1.7% of needed transfers vs 0.3% of potentially avoidable transfers, *P* **=** .01) were significantly more likely to have been necessary transfers.

Table 4 shows disposition for the overall cohort, stratified by transfer necessity. For the overall transfer cohort, 17.7% were discharged home directly from the ED, 16.8% were placed in ED observation and subsequently discharged, 6.8% were placed in ED observation and ultimately admitted, and 58.7% were admitted to the hospital. Necessary transfers were admitted 71.6% of the time, with 10.5% placed in ED observation status and discharged, 6.4% placed in ED observation and ultimately admitted, and 11.5% discharged home directly from the ED. For potentially avoidable transfers, 31.3% were admitted, 30.1% were placed in ED observation and then discharged, 7.6% were placed in ED observation and subsequently admitted, and 31% were discharged directly home.

Table 5 details the service for those patients who were admitted, both for the overall cohort and stratified by transfer necessity. For necessary transfers, 26.9% went to a spine service—12.6% went to the neurosurgery service, and 16.3% went to orthopedics. Larger numbers of patients were admitted to either the trauma service (32.4%) or to general medicine (28.4%). Of patients whose transfers were potentially avoidable, 95% of admitted patients went to general medicine, while 3.3% went to cardiology and 1.7% went to oncology.

## DISCUSSION

This five-year retrospective analysis of spine transfers to a Level I trauma center shows that even while using a broad definition of necessary transfers that included obtaining an MRI at the receiving facility, nearly a third of spine transfers were deemed potentially avoidable. This represents a potentially significant opportunity to reduce costs, ease strain on overburdened and crowded tertiary-care EDs, and save some patients afflicted with spine pathology the ordeal of being removed from their friends, families, and communities.

Just 3.3% of transferred patients in this study required emergent operative repair, and 34.6% were not even admitted to the hospital, including more than one in five patients with necessary transfers by our criteria. This suggests that even among the two-thirds of patients in this study whose transfers were considered necessary, a smaller proportion had truly emergent conditions.

Those transferred spent prolonged periods in the ED, with patients classified as potentially avoidable transfers staying an average of 16.6 hours. Ample research demonstrates that such prolonged stays can cause serious harm, particularly for elderly patients who may develop delirium in ED.^9^ These transfers also occupy scarce ED beds at a time of unprecedented crowding, and adding to this crisis may place these patients and others in crowded EDs at risk of harm. The challenge of unnecessary spine transfers must also be considered within the context of specialist availability at community hospitals. Research shows that neurosurgery is one of the most difficult specialties for maintaining ED call panels, yet neurosurgical transfers can be financially beneficial for receiving physicians and hospitals.^10^ Improving transfer decision-making through interventions like telehealth consultation could help optimize this system by reducing unnecessary transfers while preserving both the financial incentives for neurosurgical evaluation and patient access to specialized care.

This study suggests that a few diagnostic categories in particular could prove fruitful in the search for unnecessary transfers. Similar to prior research, we found that transfers for C spine fractures. L spine/sacrum/coccyx fractures, and disc disorders were all more likely to be unnecessary.^2^ While having the *ICD-10* code for back pain was associated with higher odds of a potentially avoidable transfer, this likely was not the diagnosis of the sending physician and, therefore, may not provide much insight when prospectively screening need for transfer. Patients with cervical spinal cord lesions, on the other hand, were significantly more likely to have required transfer by our metric.

While these data make clear the scope of the opportunity to reduce unnecessary spine transfers, more research is needed into how we might safely differentiate necessary from unnecessary transfers. Telehealth spine evaluations have proven accurate across multiple studies in the outpatient setting: One study of 65 patients who had a telemedicine spine visit followed by an in-person visit demonstrated no change in diagnosis 94% of the time.^11^,^12^ Research on spine telehealth evaluation in the emergency setting is comparatively lacking, although the implementation of a regional telehealth system for emergency neurosurgical consultation in France appeared to lower the rate of transfers for spine pathology.^13^ In addition, multiple studies have validated the use of telemedicine for emergent evaluation of non-spine neurosurgical diagnoses.^14^ Piloting emergency telehealth evaluation focused on spine diagnoses that are high risk for unnecessary transfer is an important next step.

## LIMITATIONS

This study has several limitations. First, given its retrospective design, we relied on the accuracy of the electronic health record to identify visits and whether a spine consult was performed, which may have introduced selection bias. The outside clinician’s decision about when to transfer patients also introduced possible selection bias, as we lacked data on patients with spine complaints who were not transferred. Additionally, the study was conducted at a single, urban Level 1 trauma center, which could limit the generalizability of our findings to other settings. To mitigate classification bias, we used objective outcome criteria based on documented clinical actions rather than chart review.

The *ICD-10* codes here reflect the primary ED diagnoses at the receiving facility, rather than the diagnosis at the referring facility when the patient was transferred or a diagnosis based on subsequent chart review. This creates a risk of classification bias, as emergency physicians likely have different approaches to assigning diagnoses. Thus, it is unclear how often this diagnosis matches either the working diagnosis of the sending physician, the remote evaluation of the spine team at the time of transfer, or the patient’s final diagnosis. In addition, these *ICD-10* codes often lacked specificity; for instance, the same lumbar fracture might be coded as “Unspecified fracture of unspecified lumbar vertebra,” “Compression fracture of lumbar vertebra, level unspecified,” or “Fracture of fourth lumbar vertebra, initial encounter for closed fracture.” This limited our analysis to broader diagnostic categories.

We deliberately chose conservative criteria to address any concerns that our metric might be inflating the number of potentially avoidable transfers. However, this does run the risk of underestimating the avoidable transfer problem. In particular, the inclusion of ED MRI imaging as a marker of necessary transfer significantly increased the proportion of transfers deemed necessary. This criterion reflects the fact that many community hospitals do not have MRI available. Some community hospitals do, however, and in such cases, access to MRI imaging would not have been a barrier to treatment in place. Our surgical service criterion reflects the presence of polytrauma transfers in this cohort. Transferred polytrauma patients who received spine service evaluation in the ED may have needed transfer to a trauma center for reasons unrelated to the spine consultation. Since it is difficult to determine whether these transfers were necessary for spine evaluation or some other trauma evaluation, we deemed transfers with admission to the trauma or other surgical service to be necessary. While we did not perform formal sensitivity analyses, these conservative criteria—particularly including all MRI recipients as necessary transfers regardless of community hospital capabilities—effectively functions as a sensitivity analysis that biases toward underestimating rather than overestimating potentially avoidable transfers.

## CONCLUSION

This study demonstrates that a third or more of patients transferred emergently for spine evaluation could potentially have been cared for in their community hospital. These data highlight a potential opportunity for the healthcare system to reduce costs and improve quality of care by treating some of these patients in their communities, particularly for conditions such as cervical or lumbar spine fracture that we found were more strongly associated with potentially avoidable transfers. Further research should evaluate the effectiveness and safety of neurosurgical telehealth programs in triaging community ED patients to either be transferred or be treated in place.

## Supplementary Information



## Figures and Tables

**Table 1:** International Classification of Diseases, 10th Rev. codes associated with diagnostic groupings used in a study of interhospital transfers for neurosurgical evaluation.

Diagnostic groupings	*ICD-10* Codes
Cervical spine fracture	S12, M48.41, M48.42, M48.43, M48.51, M48.52, M48.53, M43.01, M43.02, M43.03
Cervical spine rupture, dislocation, subluxation, or sprain	S13.0, S13.1, S13.2, S13.4, S13.8, S13.9, M43.11, M43.12, M43.13, M43.3, M43.4
Cervical spine lesion	S14.0, S14.1, S14.2
Thoracic spine fracture	S22.0, M48.44, M48.45, M48.54, M48.55, M43.04, M 43.05
Thoracic spine rupture, dislocation, subluxation, or sprain	S23.0, S23.1, S23.3, M43.14, M43.15
Thoracic spinal cord lesion	S24.0, S24.1, S24.2
Lumbar spine/sacrum/coccyx fracture	S32.0, S32.1, S32.2, S32.9, M48.46, M48.47, M48.48, M48.56, M48.57, M48.58, M43.06, M43.07, M43.08
Lumbar spine rupture, dislocation, subluxation, or sprain	S33.0, S33.1, S33.2, S33.3, S33.5, M43.16, M43.17, M43.18
Lumbar spinal cord lesion	S34.0, S34.1, S34.2
Cauda equina	S34.3, G83.4
Cord compression/paralytic syndromes	G95.2, G83.8
Spinal stenosis	M48.0
Spinal infection	M46.1, M46.2, M46.3, M46.4, M46.5, C06.1
Primary/metastatic lesion	C41.2, C70, C72.0, C72.1, C79.3, C79.4, C79.5, M84.4
Disc disorders	M50, M51
Back pain	M54
Spondylolysis or spondylolisthesis, unspecified site	M43.00, M 43.09, M43.10, M43.19

*ICD-10*, International Classification of Diseases, 10th Rev.

**Table 2:** Number and percentage of clinical and demographic characteristics by transfer necessity in a study of interhospital transfers for neurosurgical evaluation.

Characteristic	Overall number (%)	Needed transfer number (%)	Potentially avoidable transfer number (%)	P value
Female sex	943 (49.2%)	587 (45.1%)	356 (57.7%)	< .001
Mean age (standard deviation)	65 (19)	65 (19)	67 (19)	.01
Race/Ethnicity				.009
Asian	38 (2.0%)	27 (2.1%)	11 (1.8%)	
Black	112 (5.8%)	76 (5.8%)	36 (5.8%)	
Hispanic/Latino	54 (2.8%)	39 (3.0%)	15 (2.4%)	
Other	90 (4.7%)	64 (4.9%)	26 (4.2%)	
Unknown	68 (3.5%)	60 (4.6%)	8 (1.3%)	
White	1,556 (81.1%)	1,035 (79.6%)	521 (84.4%)	
English speaker	1,791 (93.4%)	1,213 (93.2%)	578 (93.7%)	.72
Self-pay	109 (5.7%)	92 (7.1%)	17 (2.8%)	.001
Emergency Severity Index				< .001
1	260 (13.6%)	228 (17.5%)	32 (5.2%)	
2	1,222 (63.7%)	836 (64.3%)	386 (62.6%)	
3 or higher	436 (22.2%)	237 (18.2%)	199 (32.3%)	
Total	1,918 (100.0%)	1,301 (100.0%)	617 (100.0%)	

**Table 3:** Diagnostic groupings by transfer necessity in a study of interhospital transfers for neurosurgical evaluation.

Diagnostic grouping	Overall number (N = 1,899) (%)	Needed transfer number (n = 1,301) (%)	Potentially avoidable transfer number (n = 617) (%)	Percentage of grouping that needed transfer
Cervical spine fracture*	363 (18.9%)	225 (17.3%)	138 (22.4%)	62.0%
Cervical spine rupture, dislocation, subluxation, or sprain	8 (0.4%)	6 (0.5%)	2 (0.3%)	75.0%
Cervical spinal cord lesion*	24 (1.3%)	22 (1.7%)	2 (0.3%)	91.7%
Thoracic spine fracture	156 (8.1%)	102 (7.8%)	54 (8.8%)	65.4%
Thoracic spine rupture, dislocation, subluxation, or sprain	0 (0.0%)	0 (0.0%)	0 (0.0%)	0.0%
Thoracic spinal cord lesion	2 (0.1%)	2 (0.2%)	0 (0.0%)	100.0%
Lumbar spine/sacrum/coccyx fracture*	200 (10.4%)	107 (8.2%)	93 (15.1%)	53.5%
Lumbar spine rupture, dislocation, subluxation, or sprain	3 (0.2%)	1 (0.1%)	2 (0.3%)	33.3%
Lumbar spinal cord lesion	2 (0.1%)	1 (0.1%)	1 (0.2%)	50.0%
Cauda equina	19 (1.0%)	16 (1.2%)	3 (0.5%)	84.2%
Cord compression/paralytic syndromes	24 (1.3%)	21 (1.6%)	3 (0.5%)	87.5%
Spinal stenosis	49 (2.6%)	26 (2.0%)	23 (3.7%)	53.1%
Spinal infection	76 (4.0%)	46 (3.5%)	30 (4.9%)	60.5%
Primary/metastatic lesion	17 (0.9%)	11 (0.8%)	6 (1.0%)	64.7%
Disc disorders*	70 (3.6%)	36 (2.8%)	34 (5.5%)	51.4%
Back pain*	370 (19.3%)	226 (17.4%)	144 (23.3%)	61.1%
Spondylolysis or spondylolisthesis, unspecified site	0 (0.0%)	0 (0.0%)	0 (0.0%)	0.0%

* Percentage of visits needing transfer within the diagnosis group significantly higher than percentage of visits needing transfer not within the diagnosis group, using a chi-square test with a Benjamini-Hochberg adjustment and a false discovery rate set at 0.05.

**Table 4:** Disposition by necessity of transfer in a study of interhospital transfers for neurosurgical evaluation.

Disposition	Overall number (%)	Needed transfer number (%)	Potentially avoidable transfer number (%)
Admitted	1,125 (58.7%)	932 (71.6%)	193 (31.3%)
ED observation then admit	130 (6.8%)	83 (6.4%)	47 (7.6%)
ED observation then discharged	323 (16.8%)	137 (10.5%)	186 (30.1%)
Discharged	340 (17.7%)	149 (11.5%)	191 (31.0%)

*ED*, emergency department.

**Table 5:** Admission service requested by necessity of transfer in a study of interhospital transfers for neurosurgical evaluation.

Admission service	Overall number (%)	Needed transfer number (%)	Potentially avoidable transfer number (%)
Cardiology	11 (0.9%)	3 (0.3%)	8 (3.3%)
Medicine	514 (41.2%)	286 (28.4%)	228 (95.0%)
Neurology	31 (2.5%)	31 (3.1%)	0 (0.0%)
Neurosurgery	127 (10.2%)	127 (12.6%)	0 (0.0%)
Oncology	21 (1.7%)	17 (1.7%)	4 (1.7%)
Orthopedics	164 (13.1%)	164 (16.3%)	0 (0.0%)
General surgery	52 (4.2%)	52 (5.2%)	0 (0.0%)
Trauma surgery	327 (26.2%)	327 (32.4%)	0 (0.0%)
Vascular surgery	1 (0.1%)	1 (0.1%)	0 (0.0%)
